# Supplementary material for: Liver-directed lentiviral gene therapy corrects hemophilia A mice and achieves normal-range factor VIII activity in non-human primates
Source: Nat Commun. 2022 May 4;13:2454. doi: 10.1038/s41467-022-30102-3 (PMC9068791; doi:10.1038/s41467-022-30102-3)
Supplement: Supplementary file 1 — Supplementary Information [file 41467_2022_30102_MOESM1_ESM.docx]

**Liver-directed lentiviral gene therapy corrects hemophilia A mice and achieves normal-range factor VIII activity in non-human primates**

**Authors:** Michela Milani^1^, Cesare Canepari^1,2^, Tongyao Liu^3^, Mauro Biffi^1^, Fabio Russo^1^, Tiziana Plati^1^, Rosalia Curto^1^, Susannah Patarroyo-White^3^, Douglas Drager^3^, Ilaria Visigalli^1^, Chiara Brombin^4^, Paola Albertini^1^, Antonia Follenzi^5^, Eduard Ayuso^6^, Christian Mueller^3^, Andrea Annoni^1^, Luigi Naldini^1,2,*^, Alessio Cantore^1,2,*^

**Affiliations:**

^1^San Raffaele Telethon Institute for Gene Therapy, IRCCS San Raffaele Scientific Institute; Milan, Italy; ^2^Vita-Salute San Raffaele University; Milan, Italy; ^3^Sanofi; Waltham, MA USA; ^4^University Center for Statistics in the Biomedical Sciences, Vita-Salute San Raffaele University; Milan, Italy; ^5^Department of Health Sciences, University of Piemonte Orientale, Novara, Italy. ^6^INSERM UMR1089, University of Nantes, CHU de Nantes; 44093 Nantes, France.

*These authors jointly supervised this work

*To whom correspondence should be addressed: [cantore.alessio@hsr.it](mailto:cantore.alessio@hsr.it), [naldini.luigi@hsr.it](mailto:naldini.luigi@hsr.it)

**Inventory of Supporting Information:**

**Supplementary Tables 1-12**

**Supplementary Figures 1-8**

**Source data**

| **LV BATCH code** | | **PDE-B17084** | **PDE-B17092** | **PDE-B17139** | **PDE-B17172** | **PDE-B18025** | **PDE-B18029** | **PDE-B18049** |
| --- | --- | --- | --- | --- | --- | --- | --- | --- |
| Particles | μg p24/mL | 41.0 | 36.3 | 64.8 | 34.0 | 43.7 | 58.7 | 23.5 |
| Titer | TU/mL | 1.2x10^8^ | 1.6x10^8^ | 1.6x10^8^ | 3.1x10^8^ | 4.9x10^8^ | 4.6x10^8^ | 9.3x10^7^ |
| Infectivity | TU/ng p24 | 2.9x10^3^ | 4.4x10^3^ | 2.4x10^3^ | 9x10^3^ | 1.1x10^4^ | 7.8x10^3^ | 3.9x10^3^ |
| Transgene Activity | - | Positive | Positive | Positive | Positive | Positive | Positive | Positive |
| FVIII protein | ng/mL | 36 | 136 | 45 | 54 | 37 | 35 | 23 |
| Total DNA | μg/mL | 7.2 | 9.4 | 9.1 | 7.0 | 9.2 | 9.2 | 6.3 |
| Producer cell protein | ng/mL | 1149 | 876 | 1194 | 774 | 933 | 1003 | 602 |
| Endotoxin | EU/mL | 0.2 | <0.2 | 0.47 | 0.6 | 0.23 | <0.2 | <0.2 |
| Sterility | - | Sterile | Sterile | Sterile | Sterile | Sterile | Sterile | Sterile |
| Total volume | Total mL | 85 | 91 | 86 | 96 | 85 | 86.5 | 72 |
| ***Transgene*** | | ***coFVIII.XTEN*** | ***coFVIII.XTEN*** | ***coFVIII.XTEN*** | ***coFVIII*** | ***coFVIII*** | ***coFVIII*** | ***coFVIII.XTEN*** |

**Supplementary Table 1 Large-scale LV batches.** The table shows the results of selected quality control assays performed on the large-scale purified LV batches. EU: endotoxin units. TU: transducing units.

**
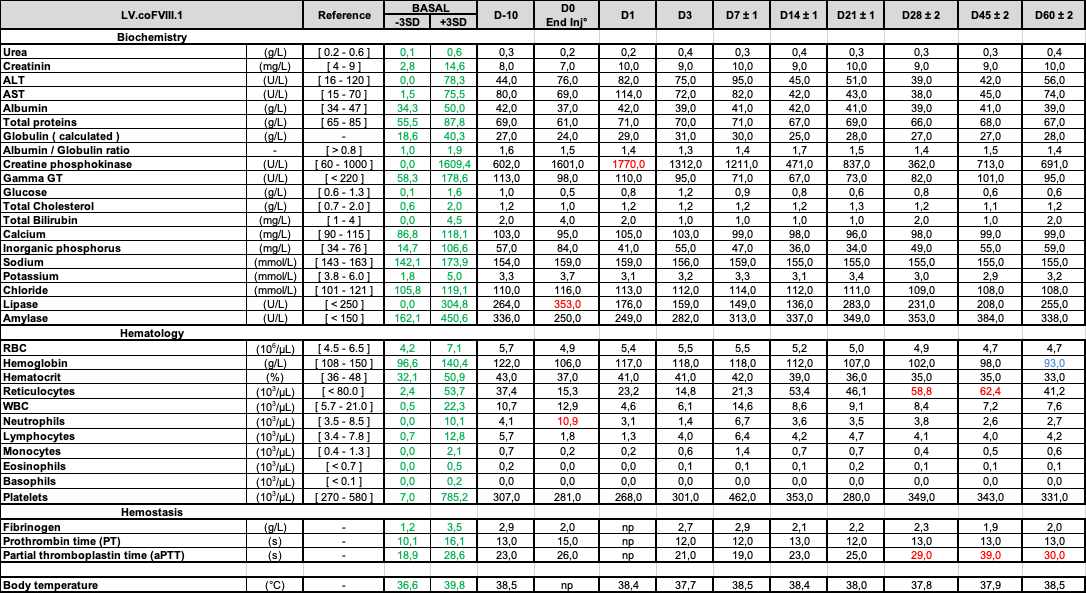
**

**
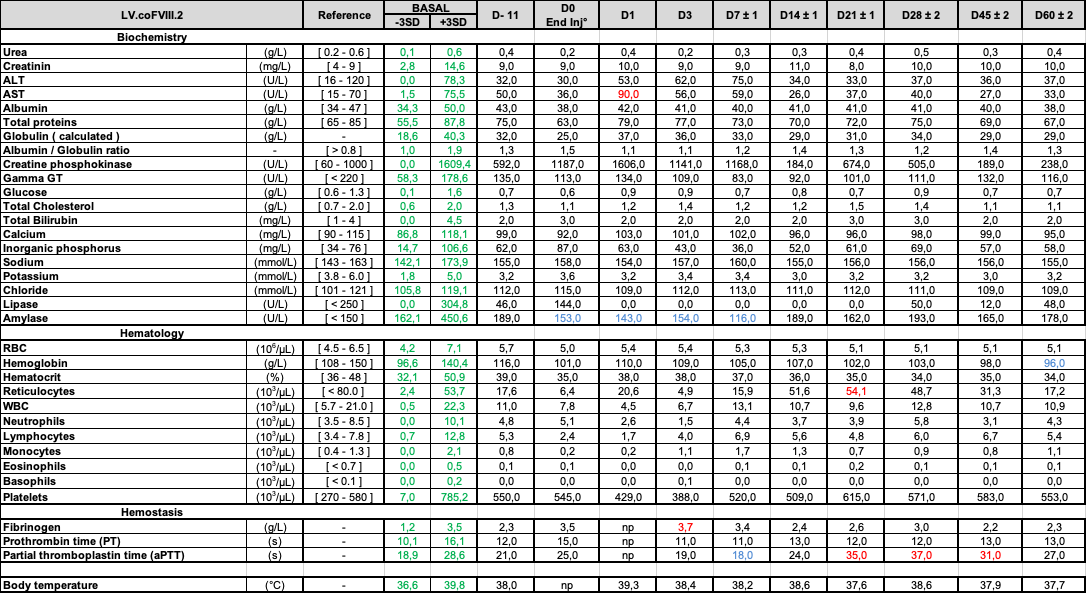
**

**
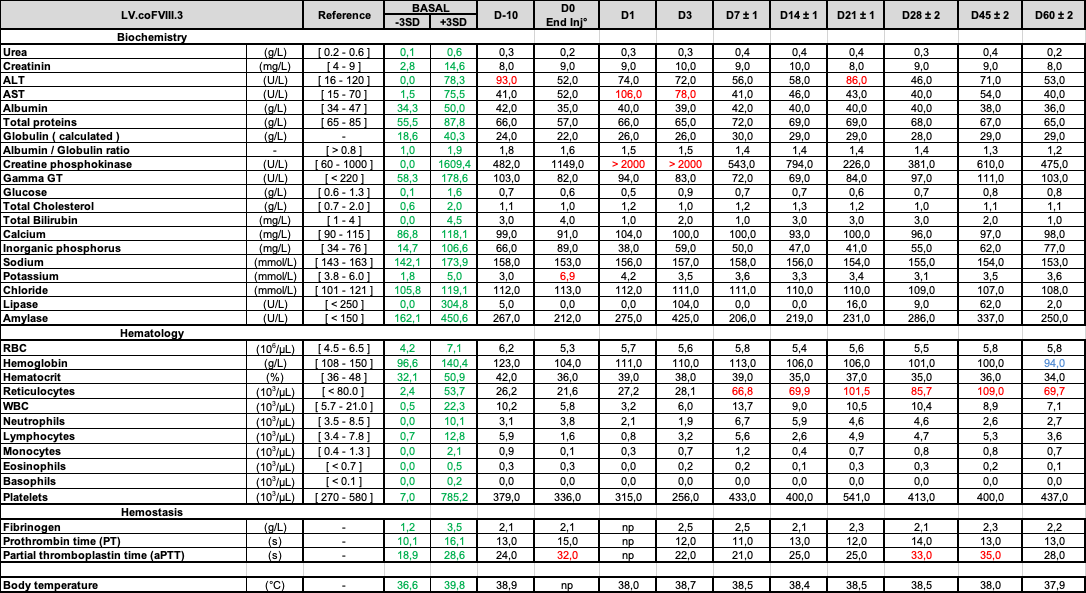
**

**
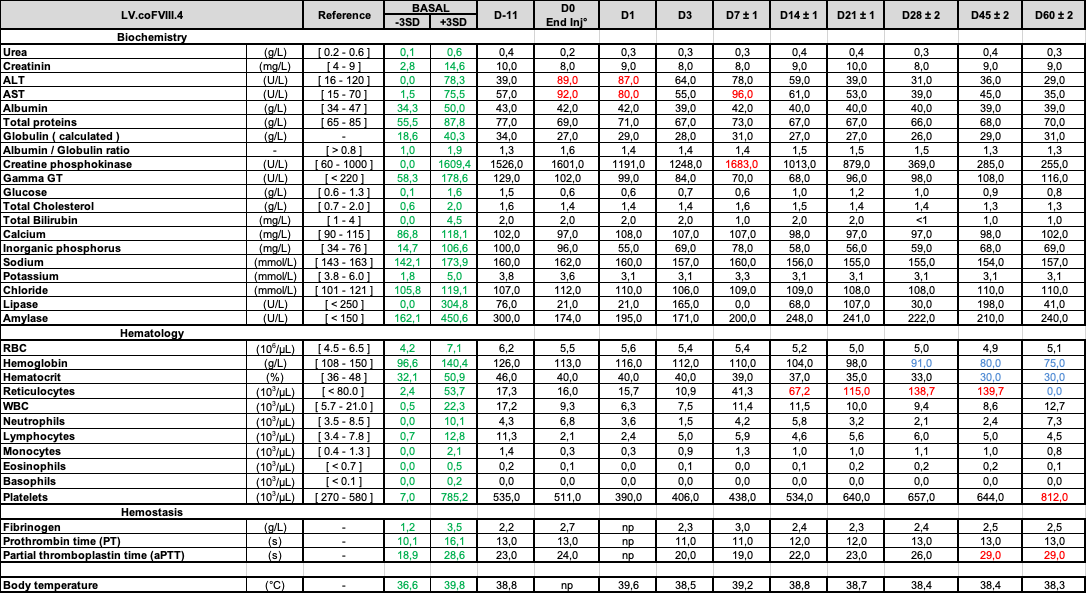
**

**
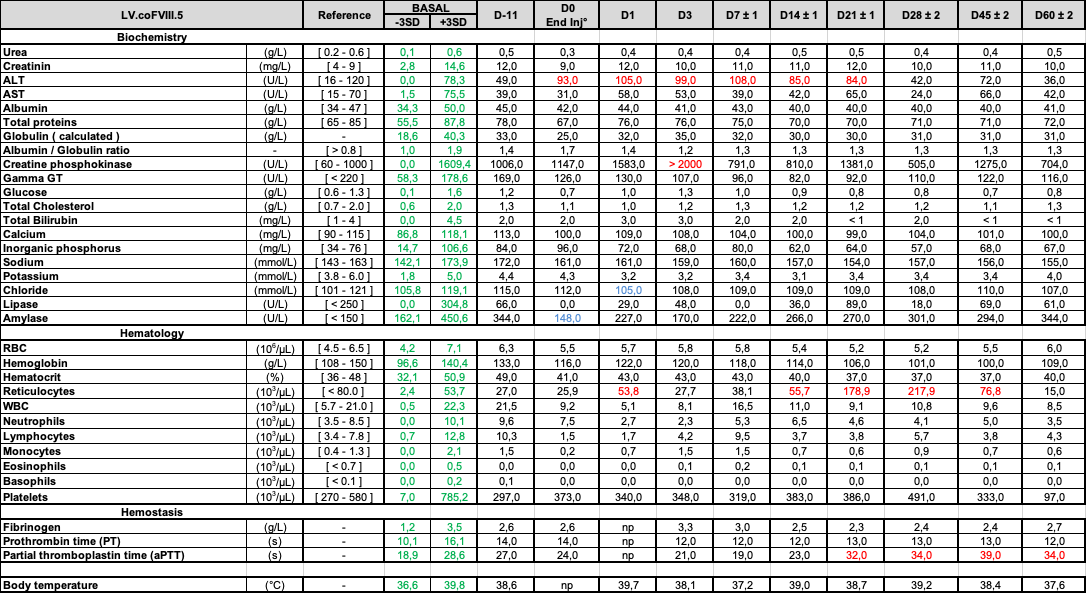
**

**
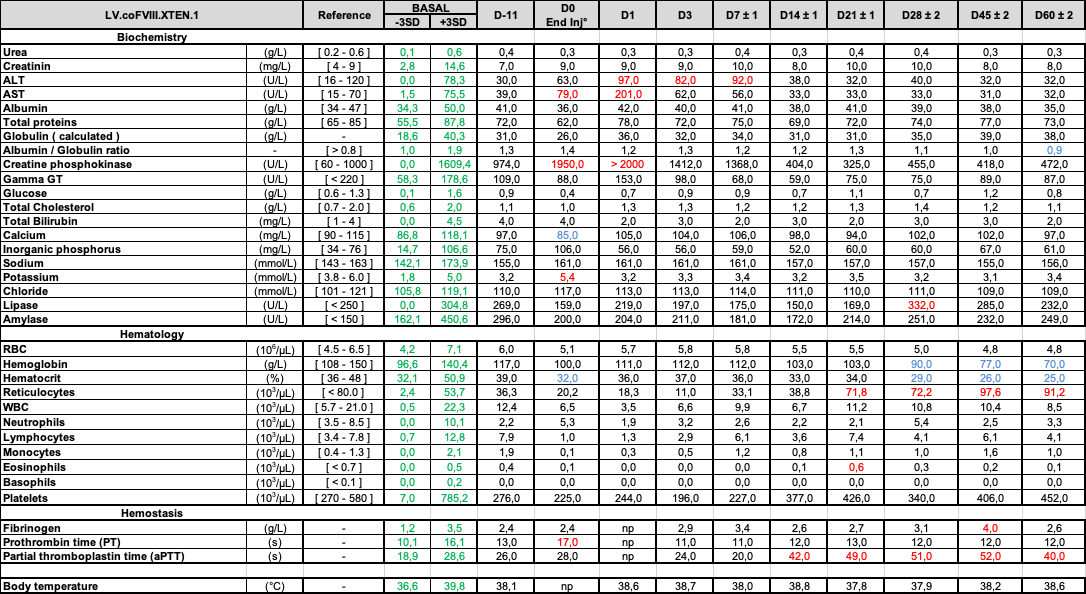
**

**
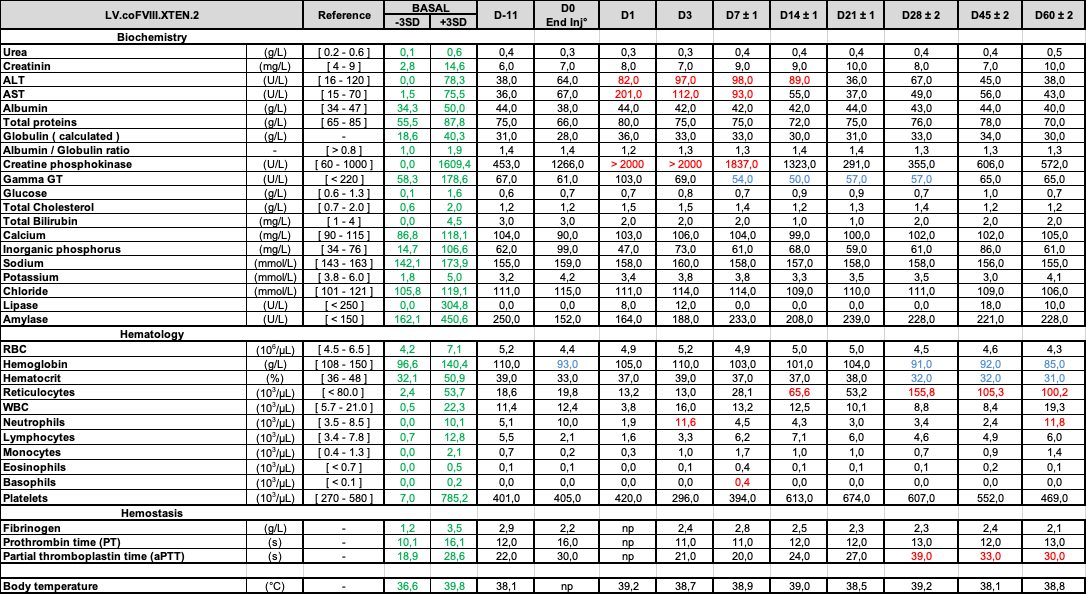
**

**
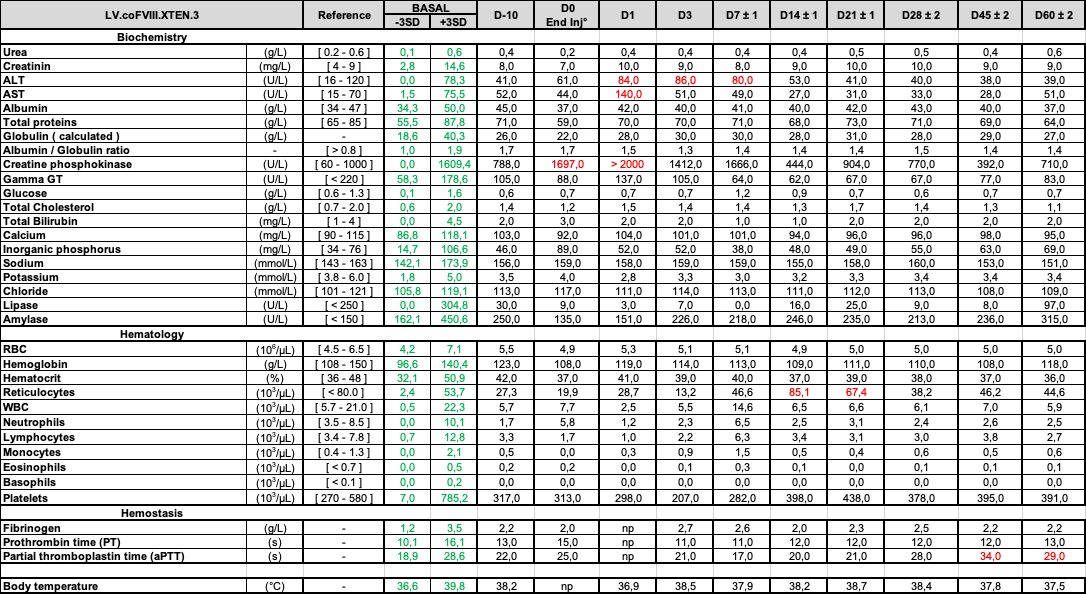
**

**
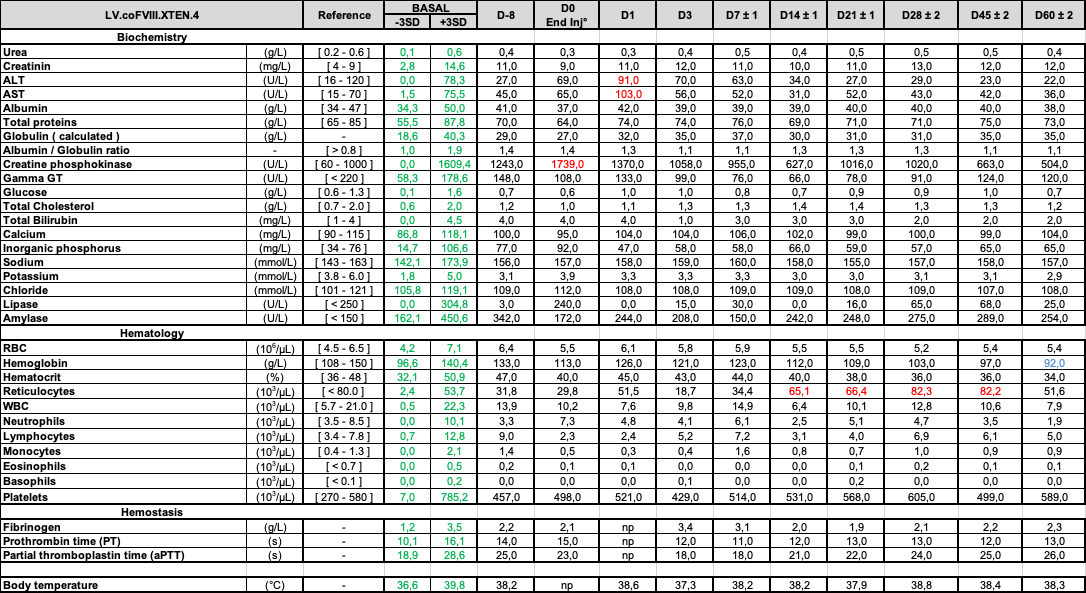
**

**
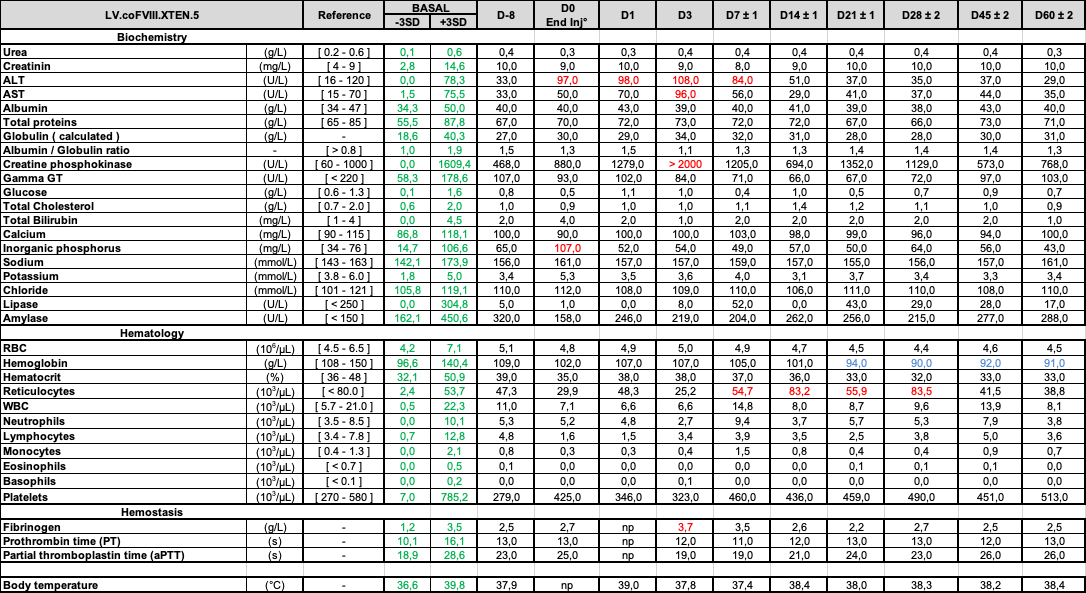
**

**Supplementary Tables 2-11 Clinical biochemistry, hematology and hemostasis following i.v. administration of LV in NHP.** The tables show the results of clinical biochemistry, hematology and hemostasis parameters in serum or plasma samples of the indicated NHP analyzed at the indicated time (days) before or after LV administration. The normal reference values for *Macaca Fascicularis* are shown in the column “Reference” and the mean±3 SD calculated on a pool of 38 pre-LV samples taken from 19 animals are shown in the column “Basal Range”. Values lower than basal range are shown in blue; values higher than basal range are shown in red. RBC: red blood cells WBC: white blood cells. Gamma GT: gamma-glutamiltranspeptidase.

**Supplementary Table 12** **Statistical analyses.** Results of LME model analysis on Fig. 1a, b and Fig. 2d, e, as indicated.

**Supplementary Figures**

**
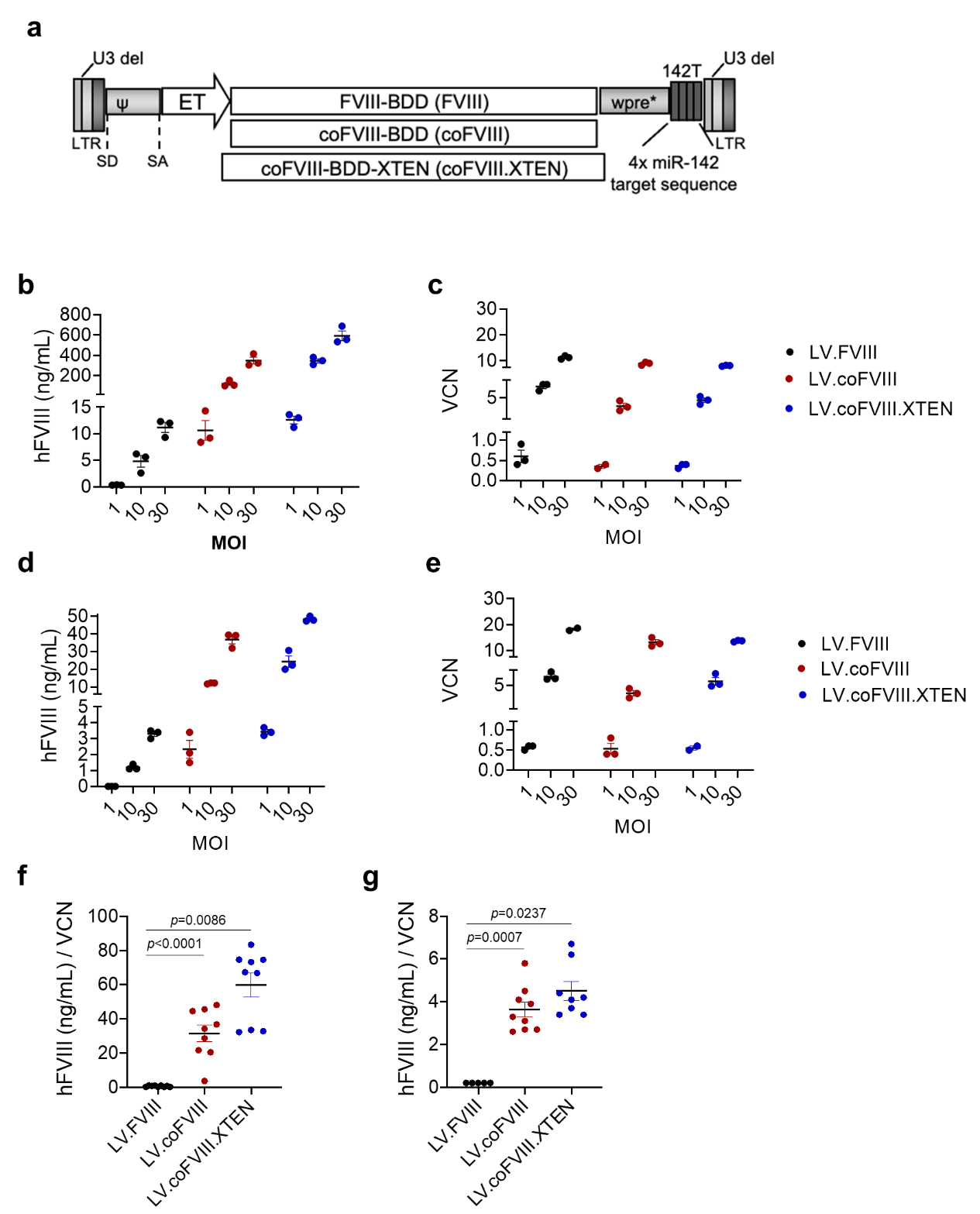
**

**Supplementary Fig. 1 FVIII transgene evaluation *in vitro* in human and murine hepatocyte cell lines.** **a** Schematic representation of the third-generation Self Inactivating (SIN) lentiviral vectors (proviral form) used in this work. U3 del: deletion of the promoter/enhancer of the HIV Long Terminal Repeats (LTR)]; SD: splicing donor site; SA: splicing acceptor site; ψ: packaging signal; Wpre*: mutated woodchuck hepatitis virus post-transcriptional regulatory element; 142T: miR-142 target sequence made of 4 tandem copies of a sequence perfectly complementary to miR-142; Hepatocyte-specific Enhanced Transthyretin (ET) promoter composed of synthetic hepatocyte-specific enhancers and transthyretin promoter. The B domain deleted (BDD), codon-usage optimized (co), and codon-usage optimized XTENylated cDNA of human factor VIII (FVIII-BDD (4374 bp), coFVIII (4374 bp), coFVIII.XTEN (4824 bp)) were used as transgenes. **b-e** Single values and mean with SEM of hFVIII measured in the supernatant (**b, d**) and VCN (**c, e**) of Huh7 cells (**b, c**) or Hepa1.6 cells (**d, e**) transduced in triplicate with the indicated LV at the indicated multiplicity of infection (MOI). 250,000 cells were seeded and supernatant was collected 72 hours post seeding. n=3 technical replicates for each tested MOI. **f, g** hFVIII output normalized on VCN in Huh7 cells (n=9 for each tested LV, **f**) or Hepa1.6 cells (n=9 for each tested LV, except for LV.coFVIII.XTEN n=8, **g**). Codon-optimized transgene output is increased at all tested doses compared to wt transgene. Kruskal-Wallis test followed by *post hoc* analysis using Dunn’s test and adjusting *p*-values with Bonferroni’s approach (only comparisons against the reference control groups have been considered).


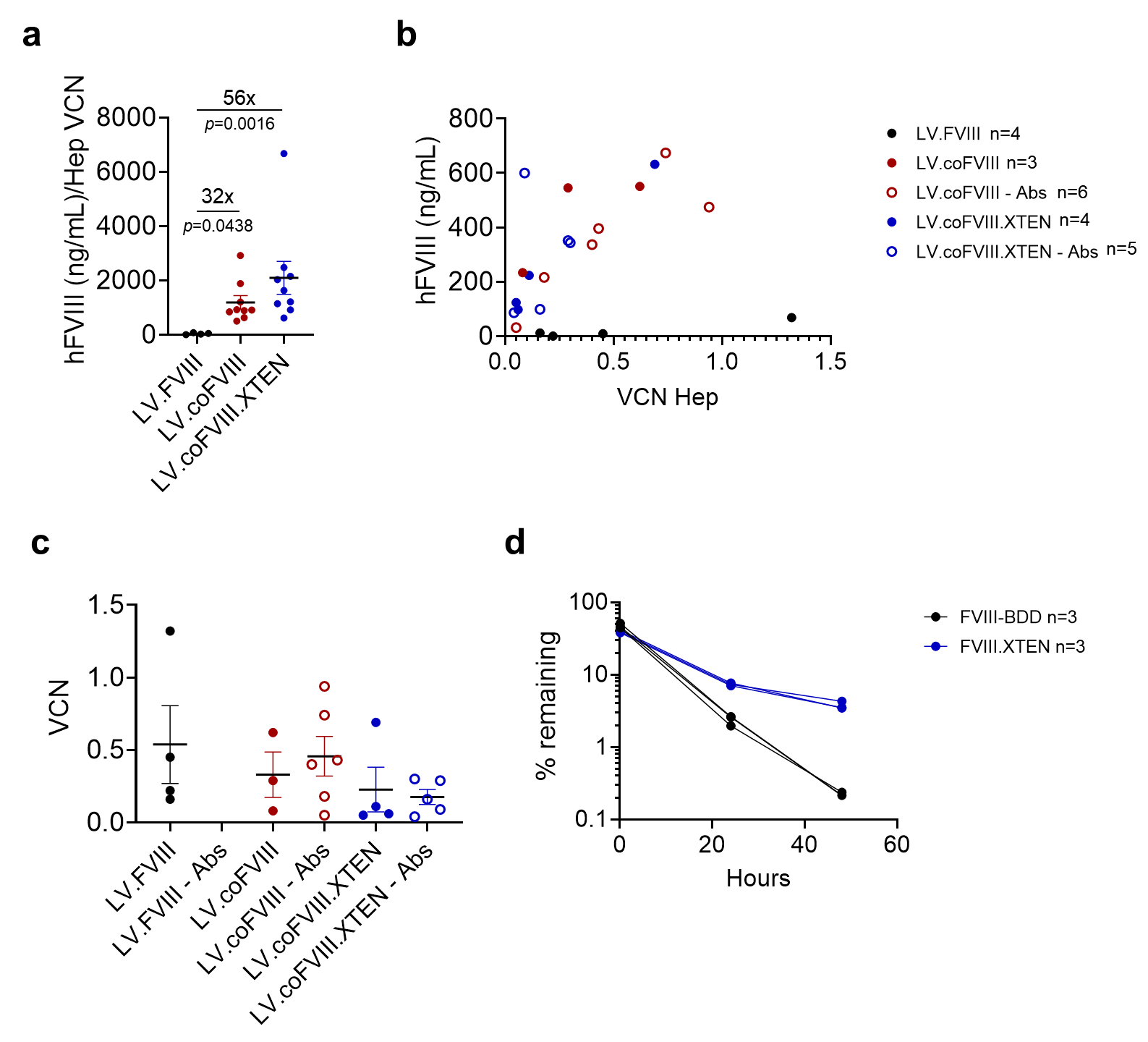


**Supplementary Fig. 2 FVIII transgene selection in HemoA mice.** **a** Single values and mean with SEM of hFVIII at plateau (mean of antigen concentration at 8-12-20-26 wks for each mouse shown in Fig. 1) normalized on VCN in purified hepatocytes (Hep). LV.FVIII n=4; LV.coFVIII n=9; LV.coFVIII.XTEN n=9. Codon-optimization of FVIII transgene allowed 32-fold increase in FVIII output *per* LV copy for LV.coFVIII and 56-fold increase for LV.coFVIII.XTEN compared to LV.FVIII. Kruskal-Wallis test followed by *post hoc* analysis using Dunn’s test and adjusting *p*-values with Bonferroni’s approach (only comparisons against the reference control groups have been considered). **b** Scatterplot displaying the relationship between hFVIII output at plateau and VCN in purified Hep. FVIII output well correlated with Hep VCN (R_S_=0.400 for LV.FVIII, *p*=0.7500; R_S_=0.800 for LV.coFVIII, *p*=0.0138; R_S_=0.767 for LV.coFVIII.XTEN, *p*=0.0369), also in mice that mice that at the end of the experiment developed anti-hFVIII Abs (empty dots), Spearman’s rank-order correlation coefficient has been computed. **c** Single values and mean with SEM of VCN in mice shown in Fig. 1. **d** Percentage of remaining FVIII activity (single values) in plasma from hemophilia A mice administered i.v. with B-domain deleted FVIII (FVIII-BDD, n=3) or XTEN-carrying BDD-FVIII (FVIII.XTEN, n=3) protein, collected at the indicated time after administration.

**
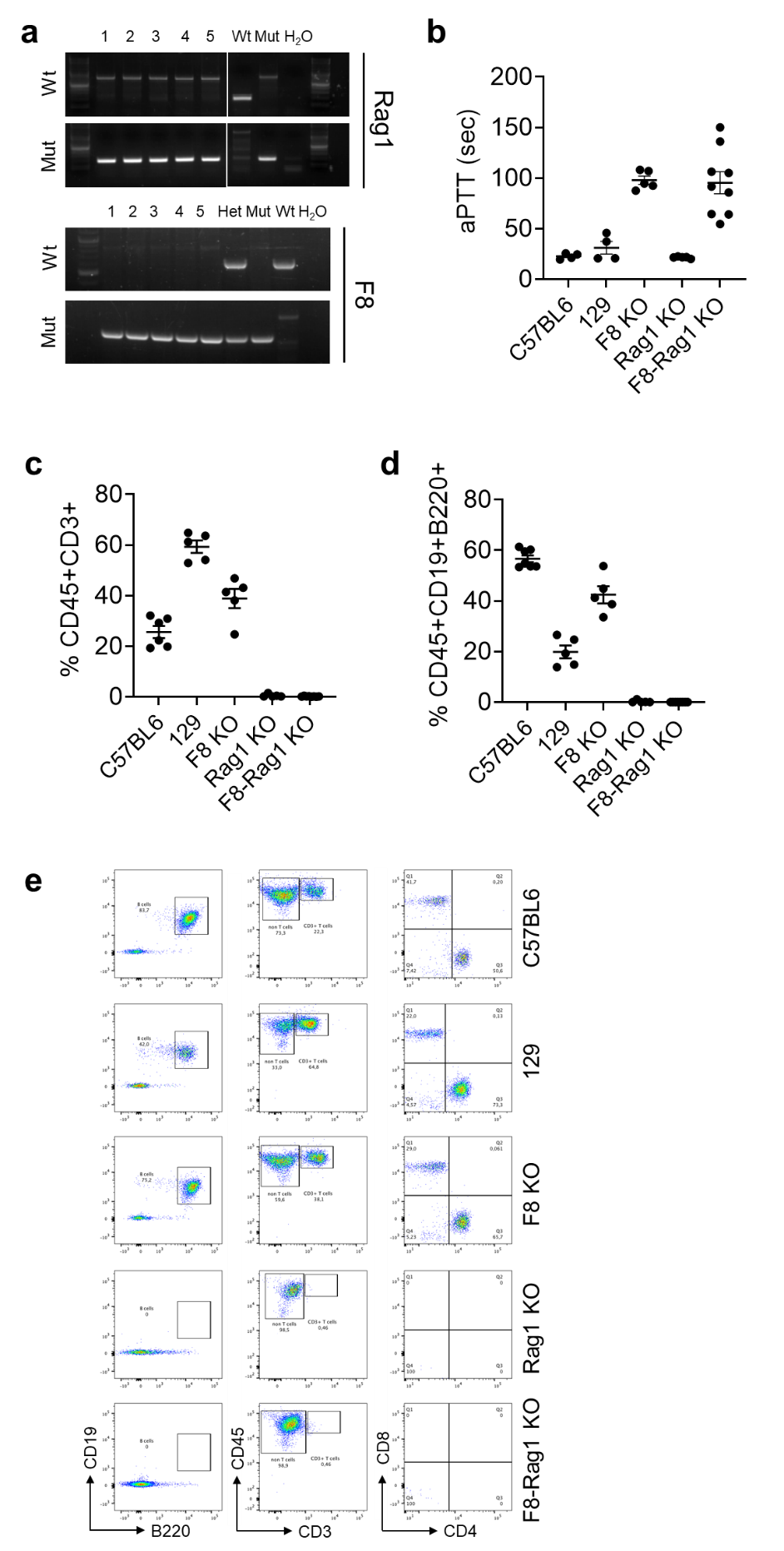
**

**Supplementary Fig. 3 Phenotype and genotype characterization of *F8*–*Rag1* double knock out (KO) mice.** **a** Representative PCR amplicons of wt or mutated (mut) *Rag1* (top) or *F8* (bottom) locus in the indicated representative mice from the colony. Expected amplicons: Rag1_wt_ = 192base pairs (bp); Rag1_mut_ = 197bp; F8_wt_ = 620bp; F8_mut_ = 420bp. A wt mouse, a known *Rag1* or *F8* KO mouse (Mut) and a heterozygous mouse (Het, available only for *F8*) are included as controls. **b** Single values and mean with SEM of activated partial thromboplastin clotting time (aPTT) measured in the plasma of *Rag1* KO (n=5), *F8* KO (n=5), *F8-Rag1* double KO (n=9) or wt mice (n=4 C57BL6, n=4 129), as indicated. **c-e** Single values and mean with SEM of T (**c**) or B lymphocytes (**d**) measured in the circulation of *Rag1* KO (n=5), *F8* KO (n=5), *F8-Rag1* double KO (n=8) or wt mice (n=6 C57BL6, n=5 129), as indicated. Representative plots and gating strategy are shown in (**e**). Source data are provided as a Source Data file.


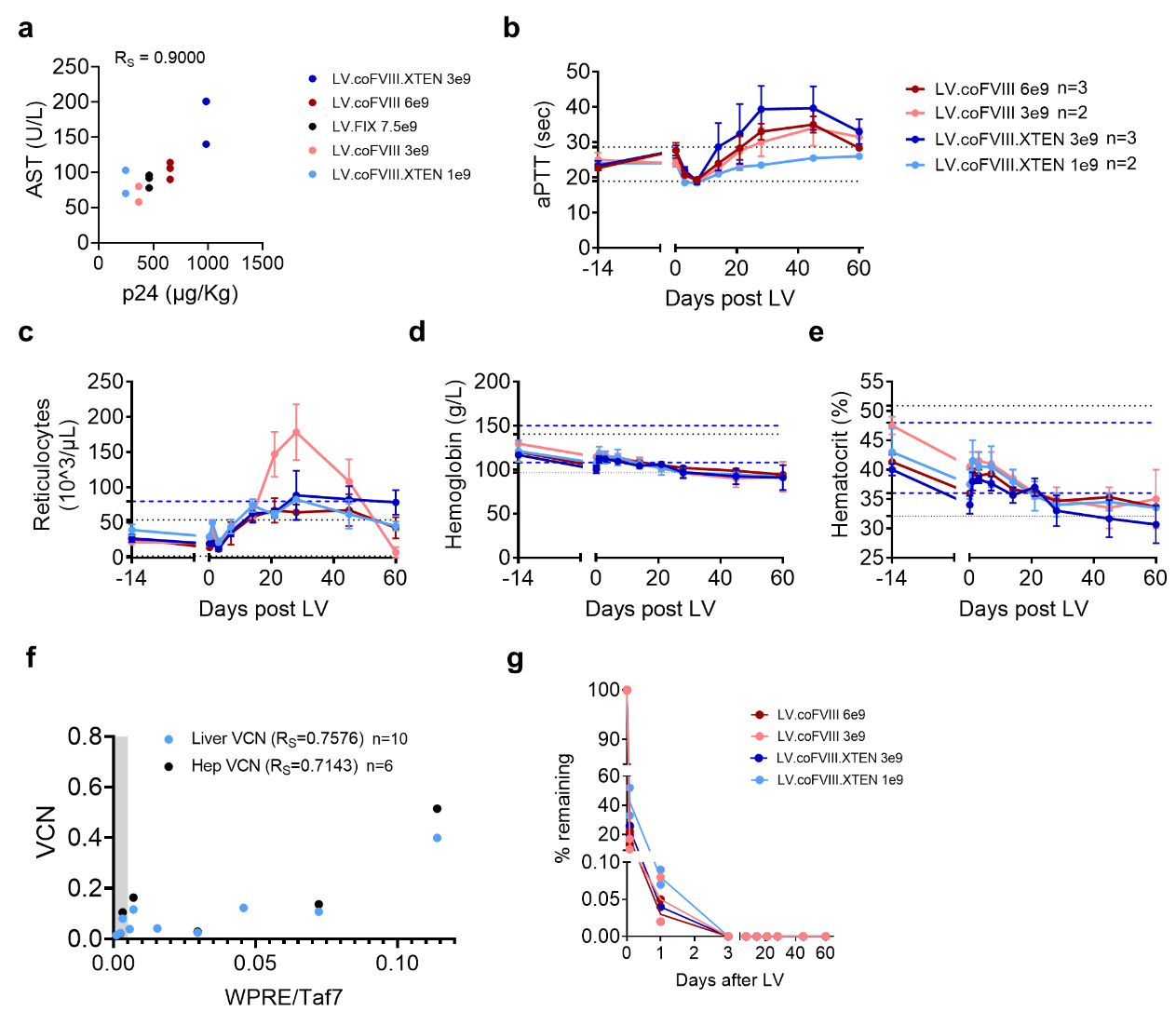


**Supplementary Fig. 4 Blood chemistry, hematology and hemostasis following *in vivo* LV gene therapy in NHP. a** Scatterplot displaying the relationship between LV particle dose (measured as HIV Gag p24 µg/Kg, X axis) and serum concentration of AST (Y axis, R_S_=0.9, *p*=0.0833) at 24 hours after LV administration in LV-treated NHP, as indicated (NHP were treated with LV.coFVIII (n=3 6x10^9^ TU/kg; n=2 3x10^9^ TU/kg), or with LV.coFVIII.XTEN (n=3 3x10^9^ TU/kg; n=2 1x10^9^ TU/kg) or with LV.FIX (n=3 7.5x10^9^ TU/kg)). Spearman’s rank-order correlation coefficient has been computed. **b-e** Mean with SEM (n=3) or range (n=2) of aPTT (**b**), counts of reticulocytes (**c**), concentration of hemoglobin (**d**) and hematocrit % (**e**) of NHP treated with LV.coFVIII (n=3 6x10^9^ TU/kg; n=2 3x10^9^ TU/kg), or with LV.coFVIII.XTEN (n=3 3x10^9^ TU/kg; n=2 1x10^9^ TU/kg) at the indicated time after LV administration. The black dotted lines show the mean±3SD calculated on a pool of 38 pre-LV samples taken from 19 animals; the blue dashed lines show the normal reference values for *Macaca Fascicularis*. **f** Scatterplot displaying the relationship between WPRE expression normalized on the endogenous TAF7 gene on RNA extracted from different liver lobes of LV-treated NHP shown in Fig. 5b (X axis) and their VCN (Y axis) in total liver shown in Fig. 5a (light blue dots, R_S_=0.7576, *p*=0.0149, n=10), or calculated Hep VCN shown in Fig. 5d (black dots, R_S_=0.7143, *p*=0.1361, n=6). The grey area represents mean WPRE background signal in the spleen. Spearman’s rank-order correlation coefficient has been computed. **g** Percentage of the serum concentrations of LV particles (measured as HIV Gag p24) recovered at the indicated time (hours) after administration of LV, as indicated, relative to the total amount of administered LV particles (NHP as in **b-e**).

**
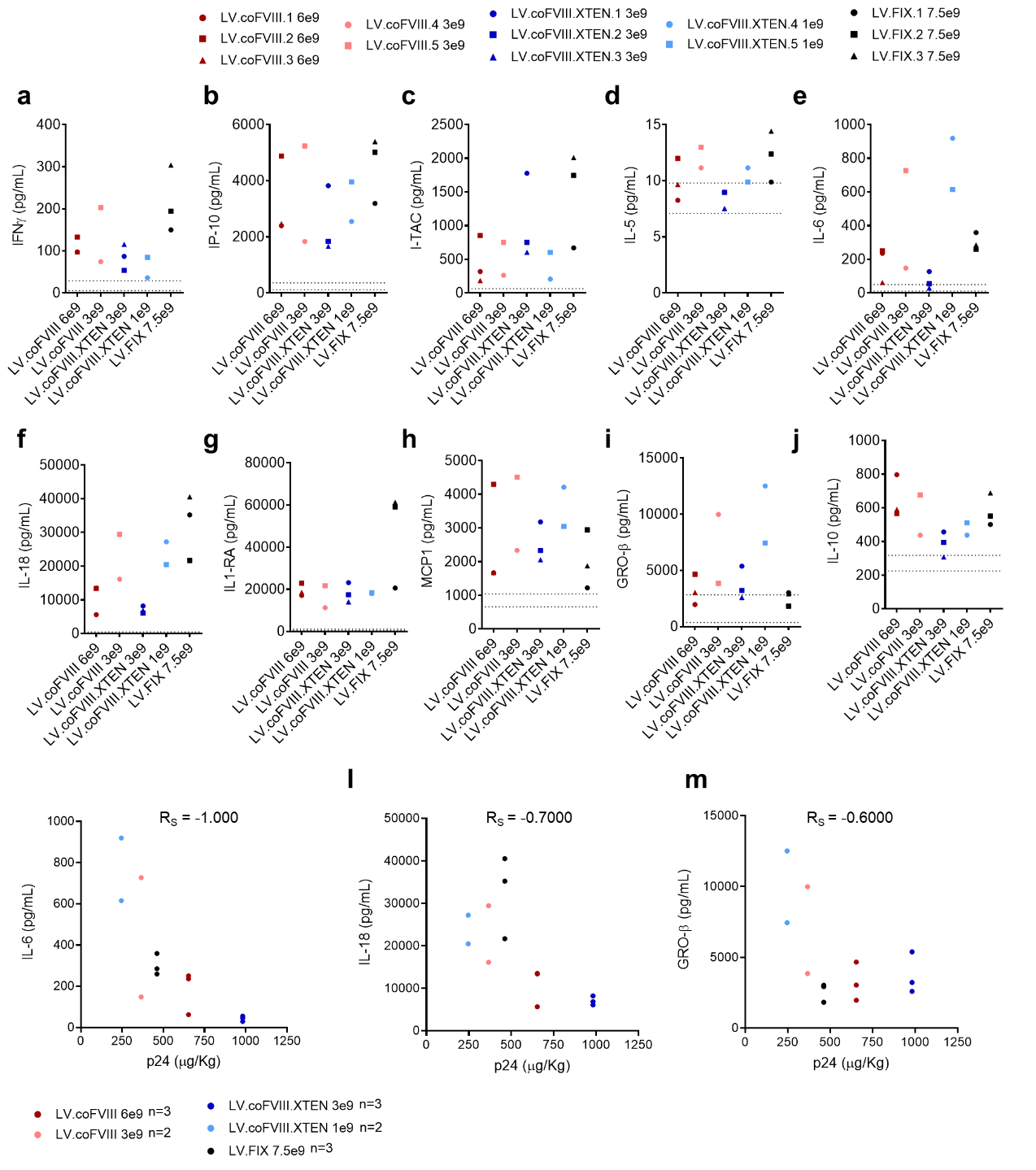
**

**Supplementary Fig. 5 Cytokine response following *in vivo* LV gene therapy in NHP.** **a-j** Single values of concentration of IFNγ (**a**), IP-10 (**b**), I-TAC (**c**), IL-5 (**d**), IL-6 (**e**), IL-18 (**f**), IL-1RA (**g**), MCP1 (**h**), GRO-β (**i**) and IL-10 (**j**) in the serum of LV-treated NHP, as indicated, at peak (24-48 hours post LV administration). Black dotted lines are mean±1SD calculated on a pool of pre-LV samples taken from 20 animals. **k-m** Scatterplot displaying the relationship between LV particle dose (measured as HIV Gag p24 µg/kg, X axis) and serum concentration of IL-6 (**k**, R_S_=-1, p=0.0167), IL-18 (**l**, R_S_=-0.7, p=0.2333) and GRO-β (**m**, R_S_=-0.6, p=0.3500) at peak in LV-treated NHP, as indicated. Spearman’s rank-order correlation coefficient has been computed.


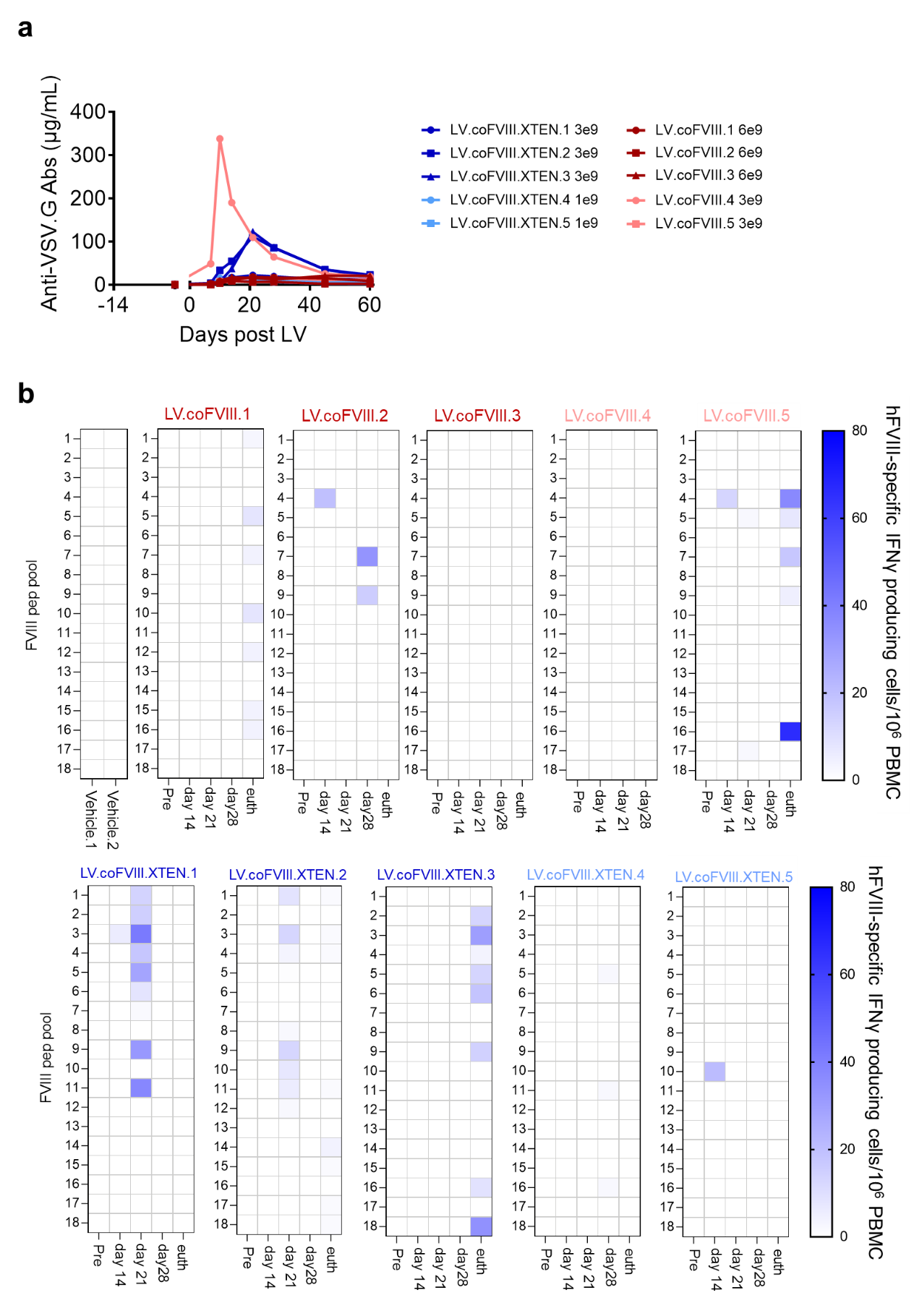


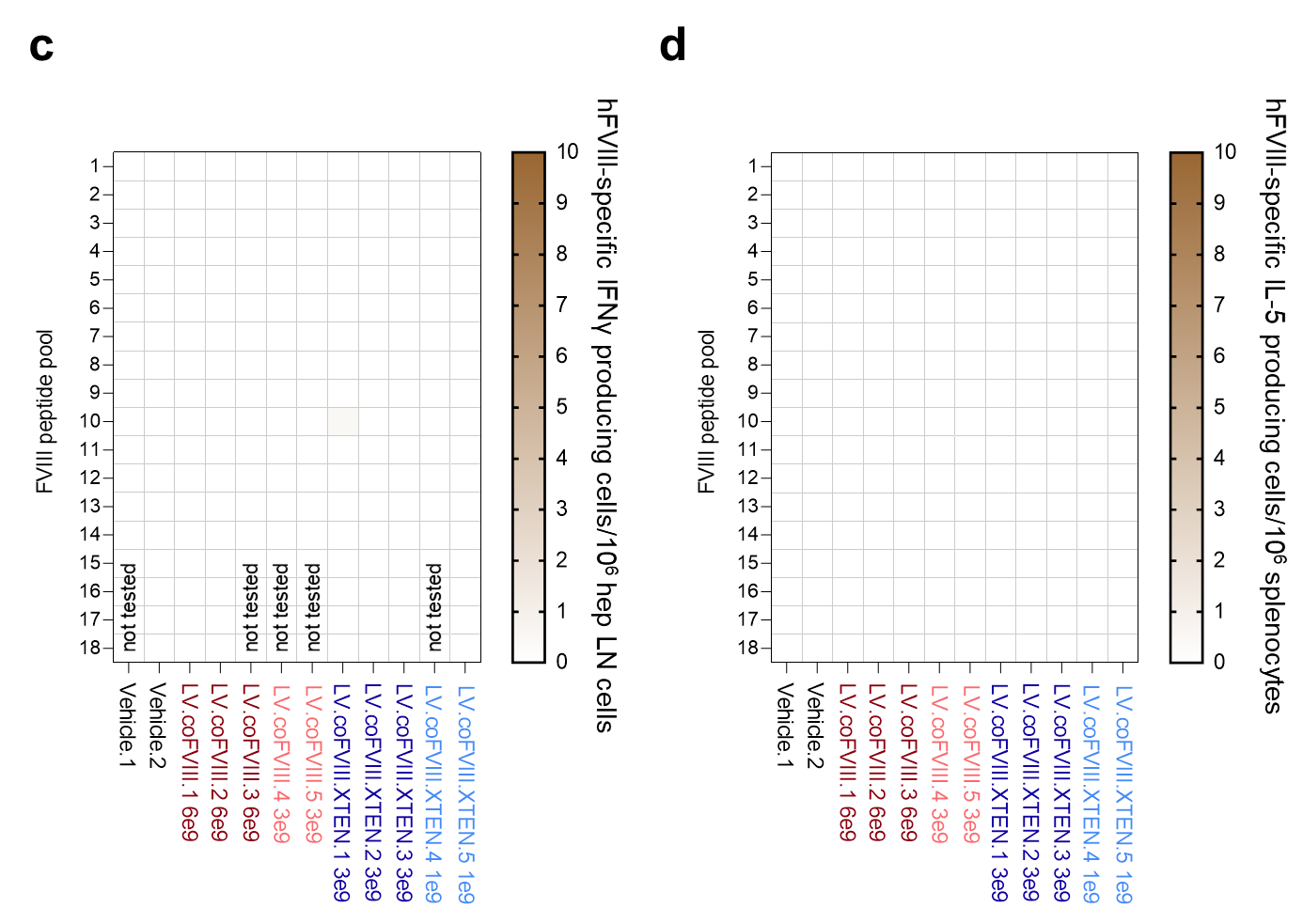


**Supplementary Fig. 6 Immune response following *in vivo* LV gene therapy in NHP.** **a** Single values of serum concentration of anti-VSV.G Abs at the indicated time after LV administration in LV-treated NHP, as indicated. **b** Heatmap reporting the frequency of hFVIII-specific IFNγ producing cells/10^6^ peripheral blood mononuclear cells (PBMC) overtime in NHP treated as indicated on top of panels. The 18 pools of FVIII peptides cover the entire transgenes encoded by administered LV. **c, d** Heatmap reporting the frequency of hFVIII-specific IFNγ producing cells/10^6^ hepatic lymph-node cells (hep LN, **c**) or IL-5 producing cells/10^6^ splenocytes (**d**) of NHP treated as indicated. Source data are provided as a Source Data file.


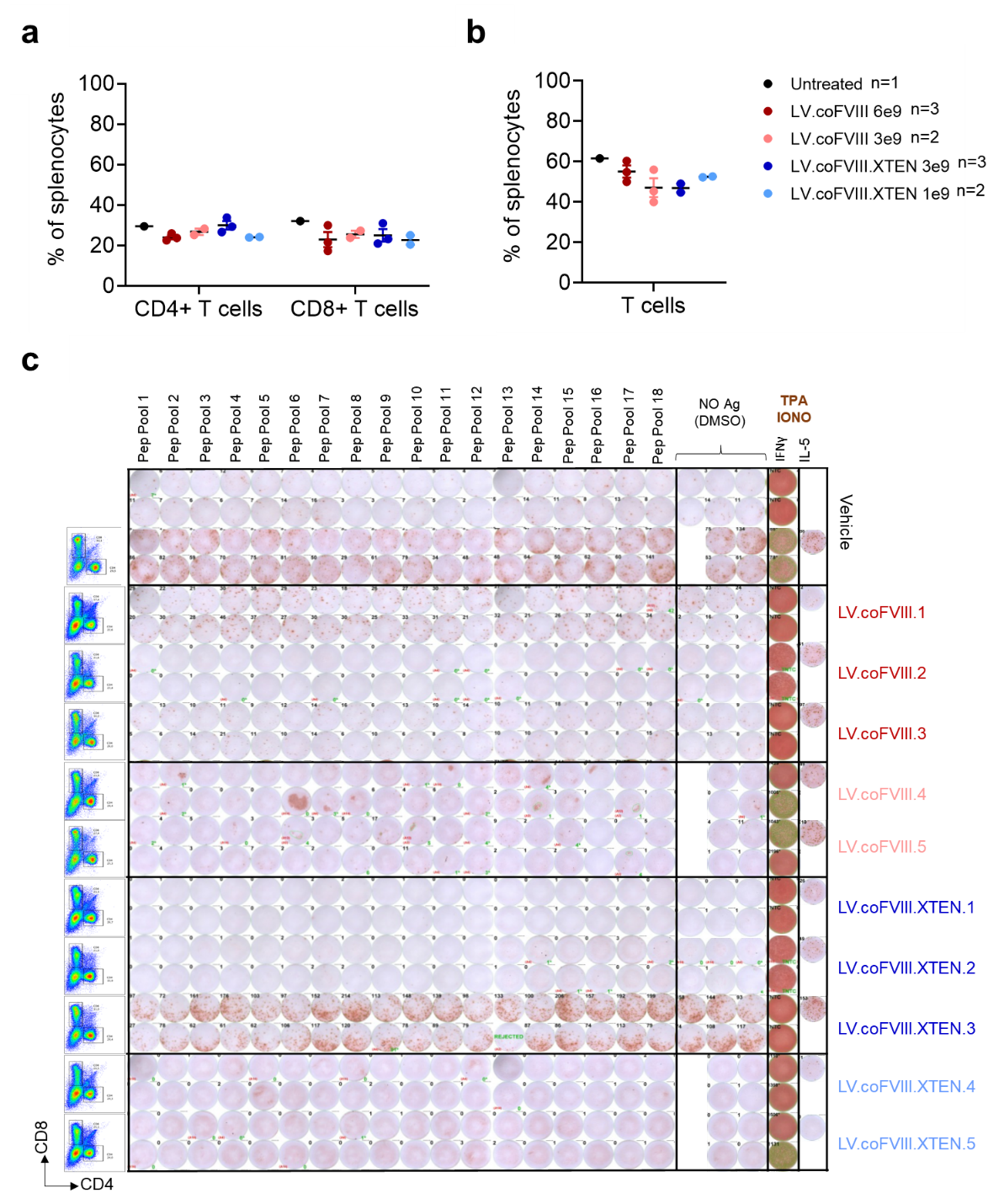


**Supplementary Fig. 7 Frequency and polyclonal stimulation of NHP splenic T cells.** **a, b** Single values and mean with SEM of the frequencies of CD8+, CD4+ (**a**) and total T cells (**b**) determined by cytofluorimetric analysis of splenocytes harvested from LV-treated NHP or vehicle control at thawing, at the time of *in vitro* functional assays (cytokine release and proliferation driven by antigenic stimulation). **c** Plots of the analysis are reported (left panels) in association of IFNγ ELISpot wells of each peptide pool stimulation (see Fig. 6 for quantification), no stimulation (no Ag, negative control) or TPA/ionomycin stimulation (positive control) from IFNγ and IL-5 ELISpot assays. Of note, IFNγ spot forming units (SFU) are too numerous to be counted (TNTC) in positive control wells, attesting the vitality and functionality of splenocytes.


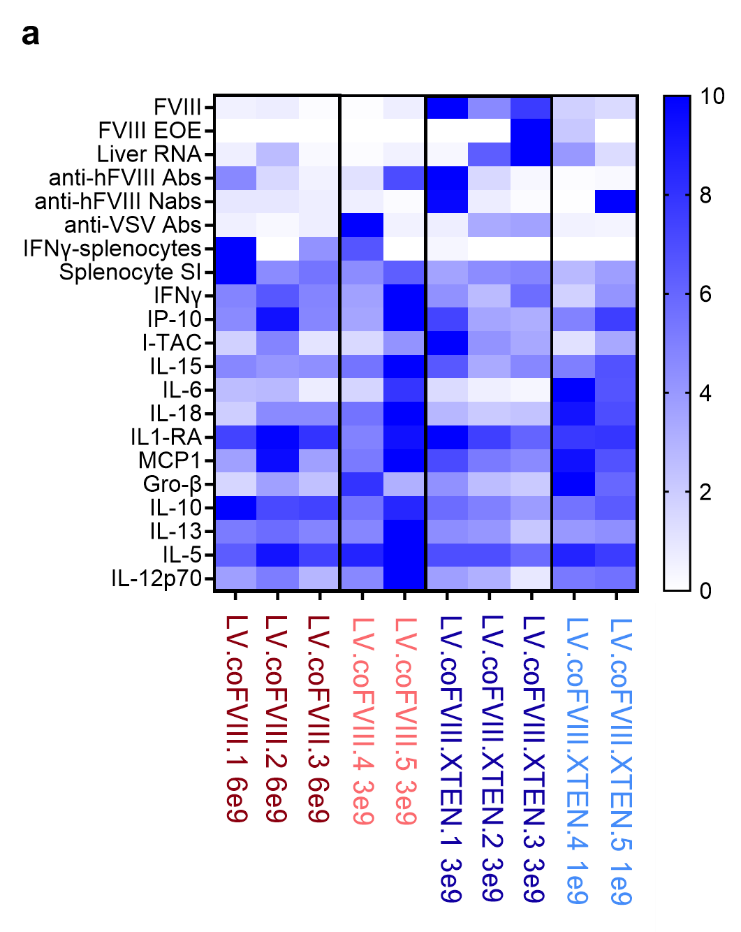


**Supplementary Fig. 8 Visual summary of the main results of *in vivo* LV gene therapy in NHP.** **a** All indicated data in NHP treated as indicated have been re-set on a scale in which the highest value measured for each parameter has been set to 10 and all the other values have been rescaled accordingly. EOE: end of experiment; Nabs: neutralizing antibodies; IFNγ-splenocytes: hFVIII-specific IFNγ producing cells/10^6^ splenocytes; SI: stimulation index. Source data are provided as a Source Data file.

**Source data**

Uncropped gels shown in Supplementary Fig. 3a.
